# Determination of KD025 (SLx-2119), a Selective ROCK2 Inhibitor, in Rat Plasma by High-Performance Liquid Chromatography-Tandem Mass Spectrometry and Its Pharmacokinetic Application

**DOI:** 10.3390/molecules25061369

**Published:** 2020-03-17

**Authors:** Jin-Ha Yoon, Thi-Thao-Linh Nguyen, Van-An Duong, Kwang-Hoon Chun, Han-Joo Maeng

**Affiliations:** Department of Pharmacy, College of Pharmacy, Gachon University, 191 Hambakmoe-ro, Yeonsu-gu, Incheon 21936, Korea; jinha89@daum.net (J.-H.Y.); linhnguyen@gachon.ac.kr (T.-T.-L.N.); anduong@gachon.ac.kr (V.-A.D.)

**Keywords:** KD025, HPLC–MS/MS, bio-analytical method, pharmacokinetics, validation

## Abstract

KD025 (SLx-2119), the first specific Rho-associated protein kinase 2 (ROCK2) inhibitor, is a potential new drug candidate currently undergoing several phase 2 clinical trials for psoriasis, idiopathic pulmonary fibrosis, chronic graft-versus-host disease, and systemic sclerosis. In this study, a bio-analytical method was developed and fully validated for the quantification of KD025 in rat plasma and for application in pharmacokinetic studies. KD025 and GSK429286A (the internal standard) in rat plasma samples were analyzed by high-performance liquid chromatography-tandem mass spectrometry with *m/z* transition values of 453.10 → 366.10 and 433.00 → 178.00, respectively. The method was fully validated according to the United State Food and Drug Administration guidelines in terms of selectivity, linearity, accuracy, precision, sensitivity, matrix effects, extraction recovery, and stability. The method enabled the quantification of KD025 levels in rat plasma following oral administration of 5 mg/kg KD025 and intravenous administration of 2 mg/kg KD025 to rats, respectively. Our findings suggest that the developed method is practical and reliable for pharmacokinetic studies of KD025 in preclinical animals.

## 1. Introduction

Rho proteins are members of the small GTP-binding protein family and operate via their downstream mediators, Rho-associated protein kinases (ROCK) 1 and 2 [1]. These kinases play a central role in various cellular functions, such as cell motility, proliferation, adhesion, migration, and apoptosis, and therefore, their potential therapeutic applications are substantial [2]. ROCK inhibitors are newly developed drugs that affect cellular functions by inhibiting the ROCK pathway. They have been demonstrated to have beneficial effects in numerous diseases, such as cardiovascular diseases [3], pulmonary diseases [4], ocular diseases [5], gastrointestinal diseases [6], depression [7], cerebral cavernous malformation [8], Alzheimer’s disease [9], idiopathic pulmonary fibrosis [10], intestinal fibrosis [11], and cancer [12,13], in which improper regulation of ROCK activity is crucial for disease pathology. Various ROCK inhibitors (> 170) have been developed so far, such as fasudil (approved for cerebral vasospasm treatment in 1995) [14], ripasudil (approved for glaucoma treatment in 2014) [15], Y-27632, Y-32885, and Y-39983 [16,17,18]. However, isoform selectivity is lacking in the majority, and this may induce severe hypotension as a dose-limiting side effect [19]. On the other hand, ROCK2 isoform-selective inhibitors with minimal hypotensive effects are considered a potential breakthrough for systemic applications [2,20]. It should be noted that ROCK2 is the dominant isoform in brain, heart, and smooth muscle cells [21], and, thus, inhibitors that specifically target ROCK2 are desirable in the context of neurodegenerative diseases. 

KD025 (SLx-2119, 2-(3-(4-((1H-Indazol-5-yl)amino)quinazolin-2-yl)phenoxy)-*N*-isopropylacetamide) was the first specific ROCK2 inhibitor to be described in 2006 and appears to be 100-fold more selective for ROCK2 than ROCK1 and considerably safer than dual ROCK inhibitors [16,22]. Upon administration, KD025 binds to and thereby inhibits the serine/threonine kinase activity of ROCK2. Furthermore, KD025 has been reported in various studies to be a potential candidate for the treatment of obesity, insulin resistance [23,24,25,26], rheumatoid arthritis, systemic lupus erythematosus [27], inflammatory bowel disease [28], chronic autoimmune disorders, chronic graft-versus-host disease [29], and fibrosis [30], as well as protection of the blood–brain barrier during thrombolysis [31]. In several phase 1 clinical trials in healthy volunteers, oral administration of KD025 was well tolerated and produced no significant adverse events, including cardiovascular side effects [19,32]. KD025 has been subjected to several phase 2 clinical trials targeting psoriasis, idiopathic pulmonary fibrosis, chronic graft-versus-host disease, and systemic sclerosis [21]. In a phase 2 study of 38 patients with psoriasis (NCT02317627), KD025 was well tolerated, with no serious side effects after oral administration, and showed results of reduced psoriasis area, normalized skin pathology, and down-regulated serum IL-17 and IL-23 cytokines [33]. A recent phase 2 study (NCT02852967) has just been finished, which aimed to evaluate the safety, tolerability, and efficacy of oral administration of KD025 in 110 patients with moderate to severe plaque psoriasis. In addition, in another phase 2a study of KD025 (NCT02841995), 54 patients with chronic graft-versus-host disease showed clinically meaningful responses with little toxicity and reductions in corticosteroid doses [34,35,36]. Currently, two phase 2 clinical trials of KD025 are being conducted in patients with idiopathic pulmonary fibrosis (NCT02688647) [37] and systemic sclerosis (NCT03919799) to evaluate treatment effects.

Thus, KD025 has attracted much attention, and, consequently, various clinical studies are being undertaken, such as dose-dependent pharmacokinetic studies and bioequivalence studies. Until now, only one previous study has used quantitative liquid chromatography–tandem mass spectrometry (LC–MS/MS) to determine KD025 levels in mouse plasma [19]. However, the employed method was neither well described nor appropriately validated. Therefore, the development and validation of a rapid and sensitive bioanalytical method for KD025 determination in plasma is highly required. In the present study, we developed and validated a bioanalytical method for assaying KD025 levels in rat plasma, using LC–MS/MS operating in positive electrospray ionization (ESI+) mode, and assessed the applicability of the devised method to pharmacokinetic studies of oral (PO) and intravenous (IV) drug administration to rats.

## 2. Results and Discussion

### 2.1. Mass Spectrometry and Chromatography

**Figure 1:**
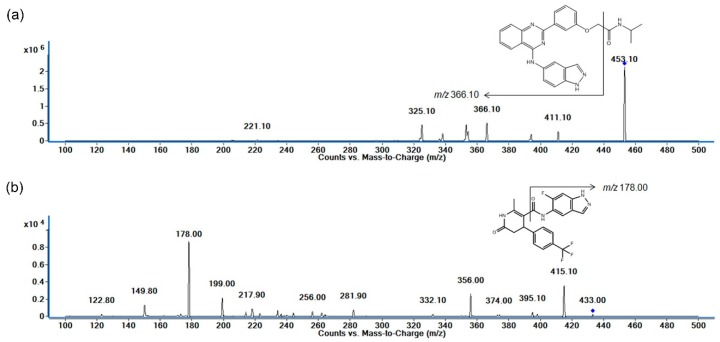
Representative product ion spectra of (**a**) KD025 and (**b**) GSK429286A (internal standard: IS) in positive ionization mode.

The chemical structures of KD025 and GSK429286A (the internal standard, IS) are depicted in Figure 1. With the ESI+ mode, protonated forms [M + H]^+^ at *m/z* 453.10 and 433.00 were chosen as precursor ions of KD025 and IS, respectively. The most prominent fragment ions in the produced ion spectra were at *m/z* 366.10 for KD025 and *m/z* 178.00 for IS (Figure 1). The transition *m*/*z* 453.10 → 366.10 for KD025 was due to the loss of the *N*-isopropyl formamide group, whereas the product ion at *m*/*z* 178.0 of IS corresponded to the separation of *N*-(6-fluoro-1H-indazol-5-yl) formamide. The fragmentor voltage and collision energy were optimized to produce maximum responses of KD025 and IS. Quantitative analyses were performed in multiple reaction monitoring (MRM) mode with *m/z* 453.10 → 366.10 for KD025 and *m/z* 433.00 → 178.00 for IS. LC conditions were then optimized to achieve symmetric peaks and adequate retention times of KD025 and IS, with separation of peaks from endogenous peaks of plasma. KD025 and IS are hydrophobic compounds; thus, several reverse phase columns were tested with different mobile phase compositions including methanol or acetonitrile. Finally, a mixture of 0.1% formic acid and acetonitrile (30:70, V/V) was employed for optimized peaks of KD025 and IS.

### 2.2. Method Validation

#### 2.2.1. Selectivity

**Figure 2:**
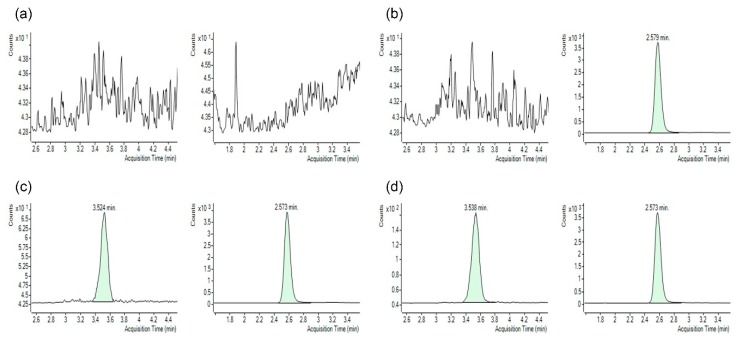

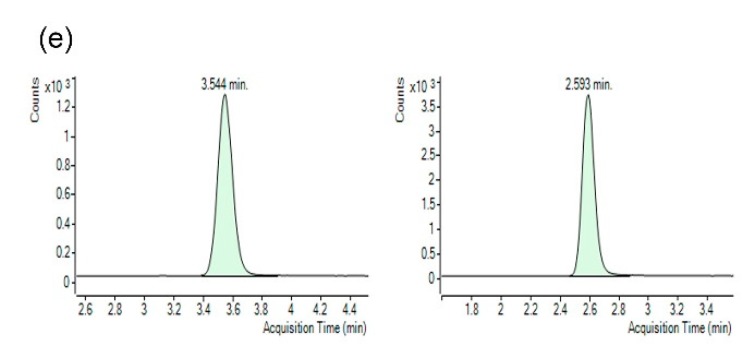
Multiple reaction monitoring LC–MS/MS chromatograms of KD025 (left) and IS (right) obtained by deproteinization of (**a**) blank rat plasma, (**b**) blank rat plasma spiked with 250 ng/mL of IS, (**c**) blank rat plasma spiked with 1 ng/mL of KD025 and 250 ng/mL of IS, (**d**) blank rat plasma spiked with 5 ng/mL of KD025 and 250 ng/mL of IS, and (**e**) rat plasma at 30 min after PO administration of KD025 at a dose of 5 mg/kg.

As shown in Figure 2, KD025 and IS were successfully separated from endogenous components of blank rat plasma using a Synergi^TM^ 4 μm polar-RP 80A column. Comparisons of chromatograms of blank rat plasma, plasma spiked with IS, and plasma spiked with KD025 and IS revealed no interference at each retention time of KD025 or IS. The peaks of KD025 and IS were symmetrical with retention times of about 3.5 min and 2.6 min, respectively. Furthermore, retention times of KD025 and IS in standard plasma samples were identical to those in plasma samples from pharmacokinetic studies at 30 min after the oral administration of KD025 to rats (Figure 2e). These results suggest that the selectivity of the devised assay was adequate for KD025 analysis.

#### 2.2.2. Linearity

Calibration curves (peak area ratio versus concentration) of KD025 in rat plasma were linear over the range of 1 to 1000 ng/mL under optimized analytical conditions. Using the weighted (1/x^2^) least squares regression analysis, the calibration equation for KD025 was y = (0.00731 ± 0.000461)x + (0.00224 ± 0.000403), with R^2^ = 0.996 ± 0.00139 (n = 5, data represent mean ± SDs; where y, x, and R refer to peak area ratio, KD025 concentration in rat plasma, and the correlation coefficient, respectively). Collectively, these results indicate that the LC–MS/MS response was directly proportional to the plasma concentration of KD025. 

#### 2.2.3. Precision and Accuracy

Quality control (QC) samples were analyzed in five replicates within one day and on five different days to determine the intra-day and inter-day accuracy/precision. Table 1 shows the accuracy and precision of five levels of KD025 in rat plasma, which were 1 ng/mL (lower limit of quantification, LLOQ), 3 ng/mL (Low QC, LQ), 40 ng/mL (Middle QC 1, MQ1), 400 ng/mL (Middle QC 2, MQ2), and 800 ng/mL (High QC, HQ). An ultra-high QC (UHQC, 8,000 ng/mL) sample, which was diluted 10 times with blank rat plasma to the concentration of 800 ng/mL (800^a^), was also investigated to evaluate the dilution integrity. The intra-day accuracy ranged from 96.6 to 100.2% for all QC samples, excluding the LLOQ sample for which the accuracy was 83.3%, and the intra-day precision was ≤ 10.8%. For the inter-day results, accuracy was in the range of 97.5% to 102.0% and precision was ≤ 10.7% for all QC samples. These results indicate that the accuracy and precision of the assay for KD025 in rat plasma were within acceptable limits, according to the United State Food and Drug Administration (US FDA) guidelines for bioanalytical method validation. In addition, the UHQC sample after dilution showed an accuracy of 101.1% and a precision of 9.7%. These observations indicate that dilution integrity of the assay was sufficient and it was reliable to quantify KD025 in samples that exceeded the upper limit of quantification, after appropriate dilution [38].

#### 2.2.4. Sensitivity

The LLOQ for the assay was established at the concentration of 1 ng/mL with an accuracy of 83.3% and a precision of 5.1%. The signal-to-noise ratio for samples at this concentration was ≥ 10. Thus, the LLOQ response is identifiable and met the acceptant criteria with an accuracy between 80% and 120% and a precision of ≤ 20% [39]. The limit of detection (LOD) calculated using the equation LOD = 3.3σ/m was 0.2 ng/mL. Experimental determination using the signal-to-noise ratio criterion (3:1) was also found to obtain the same value of LOD.

#### 2.2.5. Matrix Effect and Extraction Recovery

The extraction recovery was evaluated by comparing peak responses of KD025 in QC samples and IS with those of blanks spiked with the analytes after extraction at the same concentrations. The extraction recovery values of QC samples were > 86% for KD025 and 99.2% for IS. The matrix effect was investigated with plasma samples from three different rats (Table 2). The peak responses of QC samples, the UHQC sample, and IS (250 ng/mL) prepared with extracted blank plasma (set 2) were compared with those of standard solutions at the same concentrations (set 1) to determine the absolute matrix effect. The relative matrix effect (CV, %) was assessed by directly comparing peak responses of KD025 and IS spiked into post-extracted blank plasma samples from three different rats (set 2). The absolute matrix effect ranged from 93.3% to 102.4% for KD025 and was 100.1% for IS. For the relative matrix effect, the precision of set 2 ranged from 2.5% to 6.6% and was comparable with that of set 1. These data show that there was no notable matrix effect for KD025 and IS in rat plasma using the proposed LC–MS/MS method. 

#### 2.2.6. Stability

Stock solutions of KD025 (200 ng/mL) and IS (250 ng/mL) were stored at room temperature for 4 h or at −20 °C for 4 weeks and then evaluated for stability. After room temperature storage, observed concentrations of stored KD025 and IS were 89.6% and 100.0% of concentrations of fresh samples, respectively. After long-term storage at −20 °C, the stability was 95.4% for KD025 and 91.1% for IS. These data demonstrate that stock solutions of KD025 and IS are stable during storage for a short time at room temperature or a long time at −20 °C.

Next, the stability of KD025 was investigated in rat plasma over different typical storage/handling conditions at five QC concentrations and at the UHQC concentration (Table 3). After three freeze-thaw cycles, changes in the peak area ratios of KD025 and IS were negligible (103.3–114.3%). With regard to short-term and long-term stability, peak area ratios were similar to those of freshly prepared samples (92.8–100.5% and 95.8–109.9% for plasma samples at room temperature and at −20 °C, respectively). In addition, post-preparative stability of samples stored in an autosampler at 4 °C showed changes of 98.1–112.1% in the peak area ratios. These results suggest that KD025 is stable during typical processing, sample-handling, and storage conditions.

### 2.3. Applicability of the Assay to Pharmacokinetic Studies

**Figure 3:**
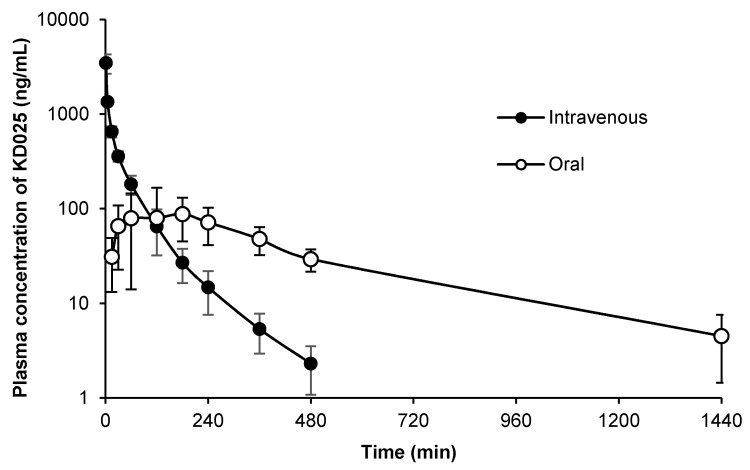
Mean plasma concentration *vs.* time profiles of KD025 after a single oral administration of 5 mg/kg KD025 (n = 5) (black circle) and an intravenous injection of 2 mg/kg KD025 (n = 5) (white circle) to rats. Results are presented as means ± standard deviations.

To determine the applicability of the assay to pharmacokinetics studies in preclinical animals, the developed method was applied to pharmacokinetic studies after single oral (5 mg/kg) and IV (2 mg/kg) administrations of KD025 to rats. The plasma concentration–time profiles of KD025 in rats are presented in Figure 3. KD025 concentrations in plasma were readily quantifiable with samples collected up to 1440 min after oral administration or 480 min after IV injection. Calculated pharmacokinetic parameters such as C_max_ (maximum plasma concentration), T_max_ (time to reach C_max_), T_1/2_ (terminal elimination half-life), AUC_last_, AUC_∞_ (area under the curve), MRT (mean residence time), CL (systemic clearance), V_ss_ (steady-state volume of distribution), and bioavailability are summarized in Table 4. Upon IV and oral administrations, AUC_∞_ values were 48.7 and 52.6 μg∙min/mL, respectively. AUC_last_ to AUC_∞_ ratios were calculated to be 94.0% and 99.2% for oral and IV administration, respectively, indicating that our LLOQ was low enough to measure exact terminal phase concentrations of KD025. In general, KD025 was rapidly eliminated (CL = 38.9 mL/min/kg) with a relatively large volume of distribution (V_ss_ = 1780.6 mL/kg) in rats after IV bolus injection (Table 4). The calculated absolute bioavailability was about 37%, which suggests that the oral bioavailability of KD025 is moderate in rats. To the best of our knowledge, there is no study to report in vivo pharmacokinetic parameters after single administration via IV and oral routes in rats. These observations show that the devised LC–MS/MS method is appropriate for pharmacokinetic studies, and might be useful in further investigations of KD025 effects in vivo, or of its pharmacological activity and mechanism in preclinical and clinical studies.

## 3. Materials and Methods

### 3.1. Reagents and Materials

KD025 (purity of = 99.42%) was purchased from MedChemExpress (Monmouth Junction, NJ, USA). GSK429286A (purity of > 98%) was supplied by Sigma-Aldrich (St Louis, MO, USA). HPLC-grade acetonitrile and formic acid were from Honeywell Burdick & Jackson (Muskegon, MI, USA) and Sigma-Aldrich (St Louis, MO, USA), respectively. All other analytical grade reagents were used without any further purification.

### 3.2. Instrumentation and Analytical Conditions

An LC–MS/MS system consisting of an electrospray tandem triple quadrupole mass spectrometer (Agilent 6490 QQQ) with an ESI+ Agilent Jet Stream ion source, coupled with a 1290 Infinity HPLC system (Agilent Technologies, Santa Clara, CA, USA), was used to perform the samples analysis. A Synergi^TM^ 4 μm polar-RP 80A column (150 × 2.0 mm, 4 μm, Phenomenex, Torrance, CA, USA) equipped with a guard column (SecurityGuard™, 4.0 × 3.0 mm, Phenomenex, Torrance, CA, USA) was used to optimally separate KD025 and GSK429286A (IS) from rat plasma endogenous substances. The mobile phase was a mixture of 0.1% formic acid and acetonitrile (30:70, V/V) with a flow rate of 0.2 mL/min under an isocratic condition for 6 min. Temperatures of the column and the autosampler were maintained at 30 and 4 °C, respectively. The injection volume was 2 μL. The ESI source was operated in positive mode. MRM transitions of *m/z* 453.10 → 366.10 and *m/z* 433.00 → 178.00 were applied for KD025 and IS, respectively. The working parameters of the mass spectrometer are detailed in Table 5. Data acquisition and processing were performed using MassHunter software (version A.06.00, Agilent Technology, Santa Clara, CA, USA).

### 3.3. Preparation of Standards and Quality Control Samples

KD025 at a concentration of 1 mg/ml in dimethyl sulfoxide was used as the master stock solution. Sets of standard solutions and QC samples were prepared by serial dilution of the master stock solution of KD025 in methanol. Plasma samples were prepared by spiking 10 μL of each standard solution into 90 μL of blank plasma to obtain final KD025 concentrations in plasma of 1, 2, 5, 10, 20, 50, 100, and 1,000 ng/mL. A two-fold volume (200 μL) of IS in acetonitrile solution (250 ng/mL) was then added, and samples were deproteinized by vortex mixing for 1 minute. After centrifugation at 14,000 rpm for 15 min, 100 μL aliquots of the supernatant were collected, and 2 μL samples were injected into the LC–MS/MS system. Plasma standards with KD025 concentrations of 1, 3, 40, 400, and 800 ng/mL were prepared as QC samples. All stock and working standard solutions were stored at −20 °C during the analysis.

### 3.4. Assay Validation

The LC–MS/MS method for KD025 analysis was validated according to the guidelines for the validation of bioanalytical methods issued by the US FDA (2018) [39].

In detail, the selectivity of the developed method was evaluated by comparing the chromatograms of blank plasma, plasma spiked with IS, plasma spiked with KD025 and IS, and plasma from rats after PO administration of KD025. Blank plasma samples from six rat sources were used to assess the selectivity.

The LOD was determined using the equation LOD = 3.3σ/m, where σ is the standard deviation of the intercept of the regression line, and m is the slope of the calibration curve [40,41]. It was also experimentally determined using the criterion of a signal-to-noise ratio of 3:1. The LLOQ was defined as the lowest concentration of KD025 that could be quantitatively determined with an accuracy between 80 and 120% and a precision of ≤ 20%. The signal-to-noise ratio for the LLOQ must be ≥ 10 [39].

The linearity of the assay was assessed using the KD025 plasma standards over the concentration range of 1 to 1,000 ng/mL. Calibration curves were constructed using the peak area ratios of KD025 and IS by weighted (1/x^2^) linear regression analysis. The acceptable criteria for precision and accuracy were set within a deviation of ±15%, except at the LLOQ, where the criteria were set at ± 20% [39]. A calibration curve with a correlation coefficient (R) of not less than 0.990 was considered acceptable for the linearity [41].

QC samples at five different concentrations of 1 (LLOQ), 3 (LQ), 40 (MQ1), 400 (MQ2), and 800 (HQ) ng/mL were used to assess accuracy and precision. Five replications of QC samples were analyzed within one day for the intra-day accuracy and precision. Inter-day data were examined by analyzing five replicates of QC samples on five different days. Percentage deviations from the nominal concentration, or the relative error (RE, %), were used to examine the accuracy, and coefficients of variation (CV, %) were used for the precision. The acceptance criteria were set at below ± 15% of RE for accuracy and within ± 15% of CV for precision, except at LLOQ, where they were set at ± 20% [39]. In addition, to evaluate the dilution integrity of the assay, which refers to its reliability for the quantification of samples with concentrations exceeding the upper limit, a UHQC with a concentration of 8000 ng/mL was diluted 10 times with blank rat plasma for the accuracy and precision test. The mean analyzed value (accuracy) should be within 15% of nominal, and the precision should be ≤ 15% for adequate dilution integrity [38].

The extraction recovery was assessed by comparing the peak areas of extracted samples at QC concentrations with those of blanks spiked with KD025 after extraction. The matrix effect was evaluated using three sources of blank plasma matrix to determine whether the endogenous components of plasma affect the ionization of KD025 and IS. Post-deproteinization samples and methanol solutions of KD025 at equivalence concentrations were compared in terms of peak areas [41,42].

The stability of stock solutions of KD025 and IS was assessed by comparing the peak response of freshly prepared methanol/acetonitrile solution with that of the solution stored at room temperature for 4 hours or at −20 °C for 4 weeks of KD025 (200 ng/mL) and IS (250 ng/mL). The stability of QC samples in rat plasma at low, middle, high, and ultra-high concentrations was investigated in terms of short- and long-term stability, post-preparative stability, and freeze-thaw stability. Short-term stability was assessed by holding QC samples at the laboratory condition (room temperature) for 4 h, while QC samples were stored at −20 °C for 4 weeks to evaluate long-term stability. The post-preparative stability of processed samples stored in an autosampler (4 °C) was evaluated after 24 hours. Freeze-thaw stability was tested over three repeated cycles, in which QC samples were frozen at −20 °C and then thawed at room temperature. Peak area ratio changes of < 15% between stored and freshly prepared samples were considered as the criteria for the stability of samples [41]. 

### 3.5. Application to Pharmacokinetic Study

The feasibility of the developed LC/MS–MS method was examined by performing pharmacokinetic studies of KD025 in rats. Animal experiments were performed in accordance with the guide for the care and use of laboratory animals issued by the National Institutes of Health, as described previously [43]. All experimental protocols were approved by the Animal Care and Use Committee of Gachon University (No. GIACUC-R2018014, approved on June 20th, 2018). Sprague–Dawley rats (8–9 weeks, 250–300 g, Nara Biotech, South Korea) were provided free access to food and water and maintained under 12 h light/dark cycles to adjust to the laboratory environment for a week before commencing the study. Rats were anesthetized by intramuscular injections (20 mg/kg) of Zoletil® (Vibrac, TX, USA) and cannulated by surgery to the femoral artery with polyethylene tubing (Clay Adams, NJ, USA) filled with heparinized saline (20 IU/mL) for blood sampling [37,38,40]. After the rats had recovered from surgery, KD025 in a vehicle mixture (DMSO:Cremophor EL:PEG400:DDW = 5:10:40:50) was administered to rats at a dose of 5 mg/kg and 2 mg/kg for oral and IV administration, respectively. Blood samples (220 μL) were collected at 0 (blank), 1 (IV only), 5, 15, 30, 60, 120, 180, 240, 360, 480 and 1440 (oral only) min after KD025 administration and equal volumes of saline were injected for compensation of fluid loss. Plasma was separated from other components of blood by centrifugation at 14,000 rpm for 15 min at 4 °C and then was stored at −20 °C until analysis.

For sample preparation, 100 μL of IS in acetonitrile solution (250 ng/mL) was added to 50 μL of plasma samples, followed by vortex-mixing in 1 min for deproteinization. Samples were then centrifuged at 14,000 rpm for 15 min at 4 °C, and 100 μL aliquots of the supernatant were collected for analysis, as described in Section 3.2. The plasma peak concentration (C_max_) and time to reach C_max_ (T_max_) were directly determined from each individual plasma concentration–time profile. Other pharmacokinetic parameters were calculated using WinNonlin® 3.1 software (Pharsight, Cary, NC, USA) by a non-compartmental method, as described previously [41,43]. Results are presented as means ± standard deviations.

## 4. Conclusions

The sensitive and rapid LC–MS/MS method was developed and validated for the determination and quantification of KD025 in rat plasma. The assay was found to have adequate selectivity, sensitivity, linearity, accuracy, and precision. KD025 was also stable during typical storing/handling conditions. The method was successfully applied to the pharmacokinetic study of KD025 in rats. It could also be applied to human samples in clinical trials after some minor modifications. With a straightforward sample preparation, a relatively short running time, a common mobile phase composition (acetonitrile and formic acid solution), and a broad linear range (1–1000 ng/mL), this developed bio-analytical method would be useful for the quantification of KD025 in preclinical/clinical pharmacokinetic and pharmacodynamic studies, to investigate the in vivo activity of KD025.

## Figures and Tables

**Table 1:** Accuracy and precision of the assay for KD025 in rat plasma.

Added Concentration (Plasma Sample, ng/mL)	Intra-day (n = 5)			Inter-day (n = 15)		
	Measured Concentration	Precision (CV, %)	Accuracy (%)	Measured Concentration	Precision (CV, %)	Accuracy (%)
1	0.8	5.1	83.3	1.0	10.7	98.3
3	2.9	7.1	96.6	2.9	3.4	97.5
40	40.9	7.0	102.3	40.8	3.9	102.0
400	390.2	5.8	97.5	408.1	2.4	102.0
800	764.2	3.2	95.5	801.1	5.6	100.1
800 ^a^	801.8	10.8	100.2	810.9	9.7	101.1

^a^ The ultra-high quality control (UHQC) (8000 ng/mL) sample was diluted 10 times with blank rat plasma to the concentration of 800 ng/mL for dilution integrity evaluation.

**Table 2:** Matrix effect and precision (CV, %) for KD025 and GSK429286A (IS) in rat plasma (n = 3).

Concentration (ng/mL)	Plasma		
	Absolute Matrix Effect (%) ^1^	Precision (CV, %)	Precision (CV, %)
		Set 1	Set 2
**KD025**			
1	95.6	4.7	2.9
3	102.4	5.8	5.1
40	95.1	4.3	6.6
400	93.3	4.3	4.9
800	97.3	8.5	2.5
800 ^a^	100.8	5.6	4.7
GSK429286A (IS)			
250	100.1	1.3	1.6

^1^ Absolute matrix effect, expressed as the ratio of mean peak area of an analyte added post-deproteinization (set 2) to that of standards of the same analyte (set 1) multiplied by 100; ^a^ The UHQC (8000 ng/mL) sample was diluted 10 times with blank rat plasma to the concentration of 800 ng/mL.

**Table 3:** Stability of KD025 in rat plasma (n = 3).

Concentration (ng/mL)	Stability (%)
Freeze-thaw stability (3 cycles)	
1	114.1 ± 1.5
3	108.7 ± 3.4
40	110.1 ± 6.5
400	103.3 ± 3.3
800	109.3 ± 5.3
800^a^	114.3 ± 2.2
Auto-sampler stability (24 h at 4 °C)	
1	112.1 ± 2.0
3	100.2 ± 0.2
40	102.0 ± 1.3
400	98.1 ± 2.1
800	101.1 ± 0.8
800 ^a^	103.3 ± 3.2
Short-term stability (4 h at room temperature)	
1	92.8 ± 5.0
3	93.0 ± 1.3
40	94.0 ± 1.6
400	99.0 ± 0.6
800	93.6 ± 1.8
800 ^a^	100.5 ± 2.5
Long-term stability (4 weeks at −20 °C)	
1	95.8 ± 3.7
3	104.9 ± 6.0
40	106.3 ± 3.0
400	109.9 ± 2.2
800	106.4 ± 1.1
800 ^a^	98.6 ± 0.5

^a^ The UHQC (8000 ng/mL) sample was diluted 10 times with blank rat plasma to the concentration of 800 ng/mL.

**Table 4:** Pharmacokinetic parameters of KD025 after single oral (5 mg/kg, n = 5) and intravenous (2 mg/kg, n = 5) administrations to rats (mean ± standard deviations).

Pharmacokinetic Parameters	PO	IV
T_max_ (min)	132.0 ± 78.2	-
C_max_ (ng/mL)	128.4 ± 74.7	4415.7 ± 1208.7 ^a^
T_1/2_ (min)	353.4 ± 169.3	88.3 ± 19.9
AUC_last_ (μg∙min/mL)	45.8 ± 12.7	52.3 ± 9.9
AUC_∞_ (μg∙min/mL)	48.7 ± 11.8	52.7 ± 10.0
AUC_∞_ /Dose (μg∙min/mL/[mg/kg])	9.7 ± 2.4	26.3 ± 5.0
MRT (min)	474.8 ± 224.9	46.4 ± 8.1
CL (mL/min/kg)	-	38.9 ± 6.2
V_ss_ (mL/kg)	-	1780.6 ± 286.9
Bioavailability ^b^ (%)	37.0%	-

^a^ C_max_ for IV refers to C_0_, estimated by noncompartmental analysis; ^b^ Bioavailability = AUC_∞__PO_/AUC_∞__IV_ × 100

**Table 5:** The LC–MS/MS working parameters.

Source Parameters	Value
Gas temperature (°C)	200
Gas flow (L/min)	12
Dwell time/transitions	200
Fragmenter voltage	380
Collision Energy	30
Nebulizer (psi)	35
Sheath gas temperature (°C)	350
Sheath gas flow (L/min)	11
Capillary voltage (V)	2500
Charging voltage (V)	1500
